# Efficacy of Anti-CD38 Monoclonal Antibodies for Relapsed or Refractory Multiple Myeloma in Stem Cell Transplant-Ineligible Patients Aged over 65 Years: A Propensity Score-Matched Study

**DOI:** 10.3390/hematolrep16040068

**Published:** 2024-11-18

**Authors:** Satoshi Yamasaki, Michitoshi Hashiguchi, Nao Yoshida-Sakai, Hiroto Jojima, Koichi Osaki, Takashi Okamura, Yutaka Imamura

**Affiliations:** 1 Department of Hematology, St. Mary’s Hospital, Kurume 830-8543, Japan; m-hashiguchi@st-mary-med.or.jp (M.H.); nao-yoshida@st-mary-med.or.jp (N.Y.-S.); jojima@st-mary-med.or.jp (H.J.); t-okamura@st-mary-med.or.jp (T.O.); y.imamura@st-mary-med.or.jp (Y.I.); 2 Department of Transfusion Medicine, St. Mary’s Hospital, Kurume 830-8543, Japan; k-oosaki@st-mary-med.or.jp

**Keywords:** older patients, multiple myeloma, relapsed or refractory, daratumumab, isatuximab, propensity score matching analysis

## Abstract

Background: The development of newer agents, including anti-CD38 monoclonal antibodies (mAbs), has significantly improved overall survival (OS) in patients with relapsed or refractory multiple myeloma (RRMM). However, the treatment of older patients with RRMM who are transplant-ineligible remains challenging. Methods: We retrospectively evaluated OS in 78 transplant-ineligible patients with RRMM who were aged ≥ 65 years and treated at our institution between February 2012 and November 2023. Results: Unadjusted OS was significantly longer in the anti-CD38 mAb-exposed group (i.e., those previously treated with daratumumab and receiving isatuximab plus pomalidomide and low-dose dexamethasone because of disease progression during treatment with daratumumab [*n* = 6], daratumumab plus pomalidomide and low-dose dexamethasone [*n* = 9], or isatuximab plus pomalidomide and low-dose dexamethasone without daratumumab-exposure [*n* = 14]) than in the anti-CD38 mAb-naïve group (no exposure to daratumumab or isatuximab [*n* = 49]) (*p* < 0.001). To address potential confounder factors associated with use or nonuse of anti-CD38 mAbs, we performed propensity score matching (PSM) using age, sex, performance status, and Geriatric 8 and Instrumental Activities of Daily Living scores. PSM identified 14 subjects from the anti-CD38 mAb-exposed group with baseline characteristics similar to those of 14 subjects from the anti-CD38 mAb-naïve group. After PSM, the adjusted OS was significantly longer in the anti-CD38 mAb-exposed group than in the anti-CD38 mAb-naïve group (*p* < 0.001). Conclusion: These findings provide insights into the optimal use of anti-CD38 mAbs in patients with RRMM who are transplant-ineligible and aged ≥65 years and on candidates who are appropriate for novel approaches, such as chimeric antigen receptor T-cell or bispecific T-cell engager therapy.

## 1. Introduction

Multiple myeloma (MM) is the third most frequently diagnosed hematological malignancy and the leading cause of hematological cancer-related death worldwide. The median age at diagnosis is 69 years, with approximately one-third of patients being over 75 years of age [1]. Although MM is a treatable disease, conventional treatments are not curative and the disease eventually relapses. The current goals of treatment for MM are to reduce symptoms, slow disease progression, and achieve remission [2]. Ideally, the main aim of treatment is to extend long-term survival [3].

Immunomodulatory agents, proteasome inhibitors, and anti-CD38 monoclonal antibodies (mAbs) have significantly improved overall survival (OS) in patients with MM over the past decade. However, this improvement has not been consistent, with outcomes for patients with high-risk features, such as adverse cytogenetic abnormalities [defined as gain(1q), t(4;14), t(14;16), t(14;20), or del(17p)], continuing to be unfavorable [4].

The choice of therapy for relapsed or refractory MM (RRMM) is challenging. Autologous stem cell transplantation should be considered for first relapse in transplant-eligible patients. For transplant-ineligible patients, especially those who are older with comorbidities and/or in assisted living facilities, the choice of treatment is determined by patient-related factors, disease-related factors, and refractoriness or prior exposure to drugs [5].

Two anti-CD38 mAbs, daratumumab and isatuximab, are approved in Japan for the treatment of MM. Daratumumab, a humanized mAb that targets a specific epitope on the CD38 protein (a transmembrane glycoprotein with ectoenzymatic activity and present predominantly on the surface of plasma cells), is available for patients with newly diagnosed MM. By contrast, isatuximab, which has some similarities to and differences from daratumumab in terms of mechanism of action, likely as a result of their binding to distinct non-overlapping epitopes on the CD38 molecule [6], is approved only for treatment of RRMM. Therefore, there is a gap in the evidence base that needs to be filled regarding the real-world management of older transplant-ineligible patients with RRMM. In this study, we retrospectively evaluated the efficacy of two anti-CD38 mAbs, namely, daratumumab and isatuximab, in transplant-ineligible patients with RRMM who were aged ≥65 years and had received chemotherapy.

## 2. Patients and Methods

The study protocol was approved by the institutional review board of St. Mary’s Hospital, Japan (approval number: 20-0706, date of approval 15 August 2024), and performed in accordance with our institutional guidelines and the principles of the Declaration of Helsinki. The need for informed consent was waived in view of the retrospective observational nature of the research and the anonymity of the data in our institutional electronic database. Nevertheless, we secured informed consent via the opt-out route on our hospital website (there were no requests for exclusion).

### 2.1. Patient Selection

The study inclusion criteria were as follows: documented diagnosis of RRMM based on the International Myeloma Working Group (IMWG) criteria [7]; age ≥ 65 years at the time of diagnosis; and at least one cycle of chemotherapy before RR status. The following exclusion criteria were applied: secondary cancer; smoking; dementia; and patient deemed unsuitable for participation in the study in the opinion of the research director. Patient-related and disease-related data at the time of diagnosis of RRMM were extracted from the electronic database. Patient-related data included age, sex, Eastern Cooperative Oncology Group (ECOG) performance status, Karnofsky performance status, Geriatric 8 (G8) level [8], activities of daily living (ADLs) [9] and instrumental ADL (IADL) scores [10], Charlson Comorbidity Index [11], dementia status, living arrangements, pulmonary function, renal function (considered normal if the estimated glomerular filtration rate was >60 mL/min per 1.73 m^2^ using the Modification of Diet in Renal Disease equation), serum N-terminal natriuretic peptide type B level (≥300 ng/L, in view of a value < 300 ng/L having a 99% negative predictive value for exclusion of acute congestive heart failure in older patients [12]), C-reactive protein level and IMWG Frailty Scale [13], Simplified Frailty Scale [14], Revised Myeloma Comorbidity Index (R-MCI) [15], United Kingdom Myeloma Research Alliance Myeloma Risk Profile (UKMRA MRP) [16], and Mayo risk [12] scores. Disease-related variables included International Staging System (ISS) [17] and Revised ISS [18] scores, cytogenetic abnormalities [19], and extramedullary disease. Responses were evaluated according to the IMWG criteria [20]. Refractoriness to a prior therapy was defined as disease progression during treatment or within 60 days of the last dose of chemotherapy. Disease progression was defined according to the IMWG criteria [20]. The enrolled patients were followed up until death or the end of follow-up.

### 2.2. Statistical Analysis

Frequencies, descriptive statistics, and disease and outcome variables were analyzed. Baseline demographic and clinical characteristics were compared between the anti-CD38 mAb-exposed and anti-CD38 mAb-naïve groups using Pearson’s chi-squared test, Fisher’s exact test, or the Kruskal–Wallis test, as appropriate. Continuous variables are expressed as the median (range) and categorical variables as the number (percentage). Patients previously treated with any anti-CD38 mAb (daratumumab and/or isatuximab) were propensity score-matched using the nearest neighbor method with a matching ratio of 1:1. The caliper width was equal to 0.2 times the standard deviation of the logit of the propensity score. A propensity score was calculated using logistic regression analysis based on age, sex, ECOG performance status, G8 level, and IADL score. Prediction performance was assessed by receiver operating characteristic (ROC) curve analysis. After propensity score matching (PSM), the balance of covariates was examined for statistical significance and standardized mean differences using Fisher’s exact test. OS was defined as the interval between diagnosis and death from any cause. Patients who had not relapsed, progressed, or died were censored at the date of the last follow-up. OS was calculated using the Kaplan–Meier method and compared between groups using the log-rank test. All statistical analyses were conducted using EZR (Saitama Medical Center, Saitama, Japan; http://www.jichi.ac.jp/saitama-sct/SaitamaHP.files/statmedEN.html, accessed on 1 August 2024) [21], which is a graphical user interface for R (The R Foundation for Statistical Computing, version 4.3.1; www.r-project.org, accessed on 1 August 2024), and a modified version of R commander (version 2.9-1) designed to add statistical functions. All tests were one-sided, and a *p*-value < 0.05 was considered statistically significant.

## 3. Results

### 3.1. Patient Characteristics

We extracted data for the 118 patients with RRMM who received second or further lines of chemotherapy at our hospital between February 2012 and November 2023. Twenty-seven patients aged < 65 years and 13 aged ≥ 65 years underwent autologous stem cell transplantation. The remaining 78 patients (66%) met the study inclusion criteria and were divided into an anti-CD38 mAb-exposed group (i.e., previously treated with daratumumab and receiving isatuximab plus pomalidomide and low-dose dexamethasone [IsaPd, *n* = 6] because of disease progression on daratumumab, daratumumab plus pomalidomide and low-dose dexamethasone [DPd, *n* = 9], or IsaPd without daratumumab [*n* = 14]) and an anti-CD38 mAb-naïve group (did not receive daratumumab or isatuximab [*n* = 49]).

The proportion of patients with high-risk characteristics at baseline was lower in the anti-CD38 mAb-exposed group than in the anti-CD38 mAb-naïve group (Table 1). Patients in the anti-CD38 mAb-exposed group were younger, had better ECOG and Karnofsky performance status, a lower rate of dementia, a higher G8 level, lower ADL and IADL scores, and a lower rate of frailty, were more likely to have an IMWG Frailty Scale score ≥ 2, less likely to have an R-MCI score indicating frailty, and more likely to meet the UKMRA MRP criteria for high-risk disease and to have a Mayo risk score ≥ III. The median follow-up duration was significantly shorter in the anti-CD38 mAb-exposed group than in the anti-CD38 mAb-naïve group (12 months [range, 1–65] vs. 41 months [range, 5–113], *p* < 0.001).

### 3.2. Overall Survival

**Figure 1:**
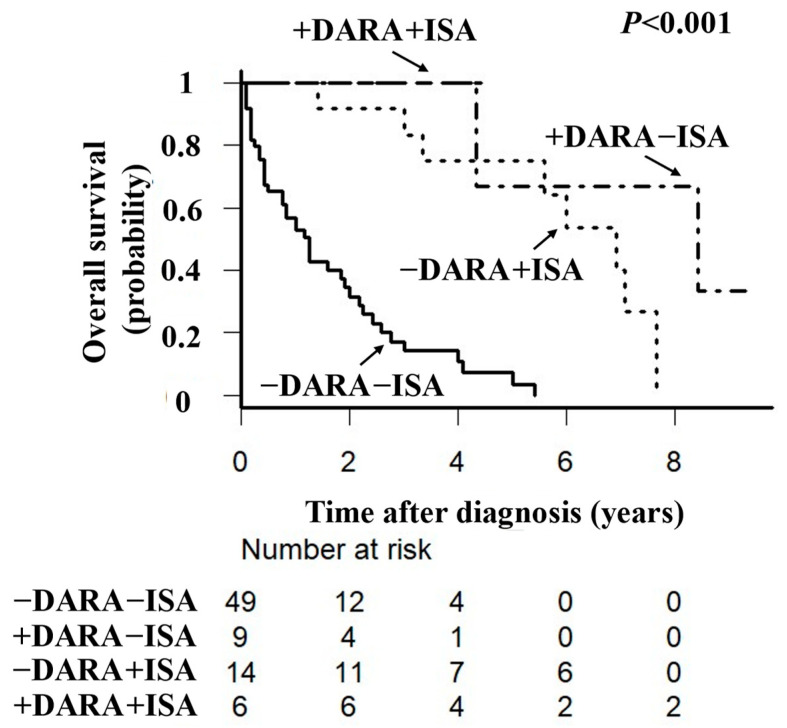
Unadjusted overall survival in older patients with relapsed or refractory multiple myeloma according to whether or not they received anti-CD38 monoclonal antibody therapy. Kaplan–Meier plots showing unadjusted overall survival in 78 patients. There was a significant difference in overall survival between the anti-CD38 monoclonal antibody-exposed and anti-CD38 monoclonal antibody-naïve groups. DARA, daratumumab; ISA, isatuximab.

**Figure 2:**
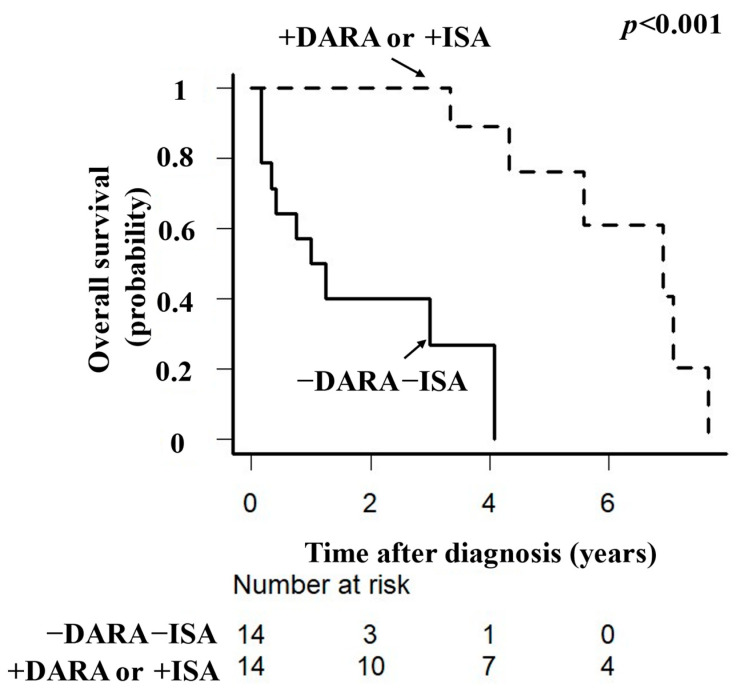
Adjusted overall survival in older patients with relapsed or refractory multiple myeloma according to whether or not they received anti-CD38 monoclonal antibody therapy. Kaplan–Meier plots showing adjusted overall survival in 78 patients. There was a significant difference in overall survival between the anti-CD38 monoclonal antibody-exposed and anti-CD38 monoclonal antibody-naïve groups. DARA, daratumumab; ISA, isatuximab.

Unadjusted OS was longer in the anti-CD38 mAb-exposed group than in the anti-CD38 mAb-naïve group (*p* < 0.001, Figure 1). PSM for age, sex, ECOG performance status, G8 level, and IADL score identified a cohort of 14 subjects in the anti-CD38 mAb-exposed group with baseline characteristics similar to those of 14 subjects in the anti-CD38 mAb-naïve group. After PSM, the area under the ROC curve was 0.825 (95% confidence interval [CI] 0.726–0.923, Figure S1). Adjusted OS was longer in the anti-CD38 mAb-exposed group than in the anti-CD38 mAb-naïve group (*p* < 0.001, Figure 2). The adjusted median 3-year OS rate was 88.9% (95% CI 43.2–98.4) in the anti-CD38 mAb-exposed group and 29.7% (95% CI 5.3–55.1) in the anti-CD38 mAb-naïve group, with respective adjusted median 5-year OS rates of 61.0% (95% CI 20.2–85.8) and 0%.

## 4. Discussion

To the best of our knowledge, this study is the first to evaluate the efficacy of anti-CD38 mAbs for RRMM in transplant-ineligible patients aged ≥ 65 years using PSM. In the unadjusted analysis, patients who received anti-CD38 mAbs had significantly better demographic and clinical characteristics, including younger age, better performance status, a lower probability of dementia, a higher G8 level, and lower ADL and IADL scores. After PSM for age, sex, ECOG performance status, G8 level, and IADL score, there were no significant differences in demographics or frailty scores between the anti-CD38 mAb-exposed group and the anti-CD38 mAb-naïve group. However, adjusted OS was significantly longer in the anti-CD38 mAb-exposed group (*p* < 0.001). Anti-CD38 mAbs are available for a small proportion of older patients with RRMM who are not eligible for transplant. However, the findings of this study demonstrate that patients with RRMM who are transplant-ineligible and aged ≥65 years might have better OS if treated with an anti-CD38 mAb.

Our main finding was that the adjusted median 3-year OS rate was 88.9% in the anti-CD38 mAb-exposed group and 29.7% in the anti-CD38 mAb-naïve group. This result indicates that anti-CD38 mAbs provide good disease control in the short term and improve survival in the longer term in patients with RRMM who are transplant-ineligible and aged ≥65 years. Aging is a complex process that leads to progressive loss of physiological integrity, organ dysfunction, increased inflammation, and susceptibility to genetic damage and epigenetic modification. Some patients in our study did not receive standard-dose chemotherapy because of their personal preference or that of the attending physician. Therefore, age and comorbidities may not have been the only reasons for not receiving anti-CD38 mAb therapy. As previously noted, this may have resulted in bias, potentially exemplified by older age and high-risk features, such as adverse cytogenetic abnormalities [defined as gain(1q), t(4;14), t(14;16), t(14;20), or del(17p)]. Given that the patients in this study were not randomized to treatment, the possibility of selection bias regarding ineligibility for anti-CD38 mAb therapy cannot be excluded.

The treatment outcomes during follow-up in our six patients with daratumumab-refractory disease were inconsistent with those previously reported [22,23], probably because of the short observation period. The outcomes in such patients require longer follow-up. Nevertheless, the treatment options after progression on anti-CD38 mAbs remain limited and outcomes are worse than after progression without anti-CD38 mAbs. The recent advent of chimeric antigen receptor (CAR) T-cell therapy [24] and bispecific T-cell engager (BiTE) therapy [25] has led to promising outcomes in patients with anti-CD38 mAb-refractory MM. Our strategy may be helpful for optimal patient selection when using CAR T-cell therapy and/or BiTE therapy in patients who have progressed after treatment with anti-CD38 mAbs. The findings of our present study may prevent unregulated use of anti-CD38 mAbs and, in the future, unregulated use of CAR T-cell and BiTE therapy.

In our study, all patients who met the eligibility criteria, which included a documented diagnosis of RRMM based on the IMWG criteria, age ≥ 65 years at the time of diagnosis, and at least one cycle of chemotherapy before RR status, in the anti-CD38 mAb-exposed group received pomalidomide and low-dose dexamethasone, which is why we could not determine the outcomes of other regimens that included anti-CD38 mAbs, except for DPd and IsaPd. Pomalidomide plus low-dose dexamethasone has been reported to have a response rate of approximately 60% in RRMM [26]. Randomized trials have demonstrated improved survival with other combinations, especially proteasome inhibitors and lenalidomide-based regimens [5]. When possible, the response and outcomes are better for triple-drug regimens, such as DPd and IsaPd, than for two-drug regimens. However, many older patients, especially those who are frail, are unable to tolerate a three-drug regimen. Prior treatment lines, refractoriness to lenalidomide, and ability to tolerate a three-drug regimen should be considered when determining treatment for older transplant-ineligible patients with RRMM.

Frailty is a consideration in older patients with MM and a predictor of treatment outcomes and toxicities [12]. In this study, we evaluated frailty using five screening tools: the IMWG Frailty Scale [12], Simplified Frailty Scale [13], R-MCI [14], UKMRA MRP [15], and Mayo risk [16] scores. After PSM, there were no significant differences in the results obtained by any of the five screening tools used for assessment of frailty between the anti-CD38 mAb-exposed group and the anti-CD38 mAb-naïve group. However, OS was significantly worse in the anti-CD38 mAb-naïve group than in the anti-CD38 mAb-exposed group. In Japan, intravenous and subcutaneous formulations of daratumumab were approved as a treatment for MM in September 2017 and March 2021, respectively, and were covered by insurance in November 2017 and May 2021. Intravenous isatuximab was approved as a treatment for RRMM in June 2020 and covered by insurance in August 2020. Almost the entire population in Japan is covered by health insurance, and it is difficult for patients to use expensive agents like anti-CD38 mAbs, including daratumumab and isatuximab, before they are covered by insurance. This explains why the older patients with RRMM in our study had not received anti-CD38 mAbs earlier in life.

Anti-CD38 mAb-containing regimens, such as DPd and IsaPd, improved OS in our older transplant-ineligible patients with RRMM. It is important to manage renal and cardiac impairment and treatment-related adverse events when attempting to prolong OS. The prognosis of MM is often worse in patients with renal impairment (RI) than in those without RI [27]. Although patients with RI are generally excluded from clinical trials, daratumumab was reported to show consistent efficacy in patients with severe RI whether or not they were on dialysis [28]. In terms of cardiotoxicity, daratumumab can be administered subcutaneously without dose overload, and inhibition of CD38 by anti-CD38 mAb therapy may reduce post-ischemic endothelial damage and have a cardioprotective effect [29]. In older patients with MM, interruptions during initial treatment owing to adverse events have a negative impact on OS [30]. Infectious diseases are one of the main reasons for premature death in patients with MM [31]. Furthermore, antibody levels have been found to be significantly lower in patients receiving daratumumab-containing regimens than in untreated patients or patients under observation only [32]. Optimal preventive strategies include vaccination against common pathogens, antibiotic prophylaxis, infection control measures, and immunoglobulin replacement. However, there are no widely accepted guidelines regarding the prevention of infection [33]. Considering the impact of infections, providing immunoglobulin replacement therapy and conventional prophylaxis may be valuable for further risk reduction.

This retrospective study had several limitations. First, it was performed at a single center, and thus the generalizability of its findings is unclear. Second, although PSM was used to reduce comparison bias, the number of factors matched was limited. Other clinical factors may play a role in OS, and their omission from PSM may have influenced our results. Our findings require confirmation in randomized controlled trials that address the limitations of PSM in terms of its inability to account for all confounders, as described previously for transplant-eligible patients with newly diagnosed MM [34]. Third, the sample size was limited because transplant-ineligible patients with RRMM who were aged ≥ 65 years needed to meet the inclusion criteria, namely, a documented diagnosis of RRMM based on the IMWG criteria and at least one cycle of chemotherapy before RR status for comparison purposes. Fourth, it was difficult to administer chemotherapy in the anti-CD38 mAbs-naïve group because of dementia. Fifth, the biology and treatment trajectories vary widely in RRMM, and it is not ideal to compare outcomes in patients with such variable prognoses and on such different chemotherapy regimens in the same study. Finally, decreased treatment intensity might have resulted in poorer outcomes in older patients. In summary, the small sample size, selection bias, and short follow-up duration might have limited our ability to analyze outcomes. Patient heterogeneity in particular may have had an unavoidable impact on our results.

## 5. Conclusions

Despite the limitations of this study, particularly its small sample size and retrospective design, which mean that its findings should be interpreted as preliminary and hypothesis-generating only, anti-CD38 mAbs might permit better OS in transplant-ineligible patients with RRMM who are aged ≥65 years. The relationship between anti-CD38 mAbs and OS requires further evaluation in large-scale prospective studies using patient-oriented outcomes, including quality of life. This strategy may be useful for selecting other novel therapies for older transplant-ineligible patients with RRMM, including CAR T-cell therapy and BiTE therapy. The results of this and future studies could facilitate shared decision-making.

## Figures and Tables

**Table 1:** Patient demographics and clinical characteristics before and after propensity score matching.

	Before Matching			After Matching		
	+DARA or +ISA	−DARA and −ISA		+DARA or +ISA	−DARA and −ISA	
**Characteristics**	*N* = 29	*N* = 49	*p*	*N* = 14	*N* = 14	*p*
**Median age (range), years**	74 (66–86)	80 (66–91)	0.003	77 (67–87)	79 (67–89)	0.534
**Age > 75 years, *n* (%)**	13 (45)	37 (76)	0.008	9 (64)	11 (79)	0.678
**Sex, *n* (%) male** **female**	17 (59) 12 (41)	29 (59) 20 (41)	1.000	8 (57) 6 (43)	8 (57) 6 (43)	1.000
**ECOG PS, *n* (%) 1** ** ≥2**	21 (72) 8 (28)	12 (24) 36 (73)	<0.001	6 (43) 8 (57)	7 (50) 7 (50)	1.000
**Karnofsky PS ≤ 70, *n* (%)**	8 (28)	35 (71)	<0.001	8 (57)	7 (50)	1.000
**Live alone, *n* (%)**	6 (21)	17 (35)	0.212	1 (7)	5 (36)	0.165
**Dementia, *n* (%)**	1 (3)	12 (24)	0.024	1 (7)	2 (14)	1.000
**Pulmonary function abnormality *n* (%)**	1 (3)	1 (2)	1.000	1 (7)	0	1.000
**Renal function (eGFR_MDRD_** **<60 mL/min per 1.73 m^2^), *n* (%)**	17 (59)	36 (73)	0.213	10 (71)	12 (86)	0.648
**CCI > 3, *n* (%)**	21 (72)	38 (78)	0.538	11 (79)	11 (79)	0.844
**G8 ≥ 10, *n* (%)**	21 (72)	11 (22)	0.001	6 (43)	7 (50)	0.792
**ADL ≤ 4, *n* (%)**	6 (21)	33 (67)	0.010	6 (43)	6 (43)	1.000
**IADL ≤ 5, *n* (%)**	18 (62)	43 (88)	0.003	13 (93)	11 (79)	0.908
**Median CRP levels (range), mg/dL**	0.10 (0–33.0)	0.20 (0–5.3)	0.360	0.1 (0–33.0)	0.1 (0.02–5.3)	0.577
**NT-proBNP levels ≥ 300 ng/L, *n* (%)**	2 (7)	10 (20)	0.193	1 (7)	1 (7)	1.000
**ISS *n* (%) II** ** III**	18 (62) 9 (31)	24 (49) 23 (47)	0.452	8 (57) 5 (36)	7 (50) 6 (43)	1.000
**R-ISS, *n* (%) II** ** III**	25 (86) 2 (7)	35 (71) 12 (24)	0.125	12 (86) 1 (7)	11 (79) 2 (14)	1.000
**EMD, *n* (%)**	1 (3)	4 (8)	0.646	1 (7)	2 (14)	1.000
**High-risk cytogenetics, *n* (%)**	2 (7)	12 (24)	0.068	1 (7)	2 (14)	1.000
**IMWG frailty scale ≥ 2, *n* (%)**	21 (72)	47 (96)	0.004	14 (100)	14 (100)	1.000
**Simplified frailty scale ≥ 2, *n* (%)**	26 (90)	45 (92)	0.703	13 (93)	13 (93)	1.000
**R-MCI, *n* (%) Intermediate fitness** ** Frail**	12 (41) 1 (3)	29 (59) 9 (18)	0.007	8 (57) 1 (7)	6 (43) 2 (14)	0.763
**UKMRA MRP, *n* (%) Medium risk** ** High risk**	19 (66) 8 (28)	16 (33) 32 (65)	0.003	6 (43) 8 (57)	5 (36) 9 (64)	1.000
**Mayo Risk Score ≥ III, *n* (%)**	7 (24)	36 (73)	<0.001	6 (43)	7 (50)	0.275
**Median *N* (range) of prior lines of therapy**	4 (1–7)	3 (1–3)	0.068	3 (1–3)	3 (1–3)	1.000
**Prior therapy, *n* (%)** Proteasome inhibitors Immunomodulatory drugs	12 (41) 29 (100)	11 (22) 49 (100)	0.125	6 (43) 14 (100)	5 (36) 14 (100)	1.000
**Best response before DARA or ISA, *n* (%)** Complete response Very good partial response	2 (7) 2 (7)	4 (8) 4 (8)	1.000	1 (7) 1 (7)	2 (14) 2 (14)	1.000

Variables were compared between the groups using Pearson’s chi-squared test, Fisher’s exact test. or the Kruskal–Wallis test as appropriate. ADL, activities of daily living; CCI, Charlson Comorbidity Index; CRP, C-reactive protein; DARA, daratumumab; ECOG, Eastern Cooperative Oncology Group; eGFR_MDRD_, glomerular filtration rate estimated by modification of diet in renal disease; EMD, extramedullary disease; IADL, instrumental activities of daily living; IMWG, International Myeloma Working Group; ISA, isatuximab; ISS, international staging system; NT-proBNP, N-terminal natriuretic peptide type B; PS, performance status; R-ISS, Revised ISS; R-MCI, Revised Myeloma Comorbidity Index; UKMRA MRP, United Kingdom Myeloma Research Alliance Myeloma Risk Profile.

## Data Availability

The institutional review board of St. Mary’s Hospital, Japan, does not allow open access. However, upon reasonable request, additional analyses can be performed after contacting the corresponding author.

## Supplementary Materials

The following supporting information can be downloaded at: https://www.mdpi.com/article/10.3390/hematolrep16040068/s1, Figure S1: Receiver-operating characteristic curve analysis showed an area under the curve of 0.825. A propensity score was calculated using logistic regression analysis based on age, sex, Eastern Cooperative Oncology Group performance status, Geriatric 8 level, and instrumental activities of daily living scores. After propensity score matching, the area under the receiver-operating characteristic curve was 0.825 (95% confidence interval 0.726–0.923).
