# Supplementary material for: Efficacy of Anti-CD38 Monoclonal Antibodies for Relapsed or Refractory Multiple Myeloma in Stem Cell Transplant-Ineligible Patients Aged over 65 Years: A Propensity Score-Matched Study
Source: Hematol Rep. 2024 Nov 18;16(4):714–23. doi: 10.3390/hematolrep16040068 (PMC11587159; doi:10.3390/hematolrep16040068)
Supplement: Supplementary file 1 [file hematolrep-16-00068-s001.zip › hematolrep-3210002-supplementary.pdf]

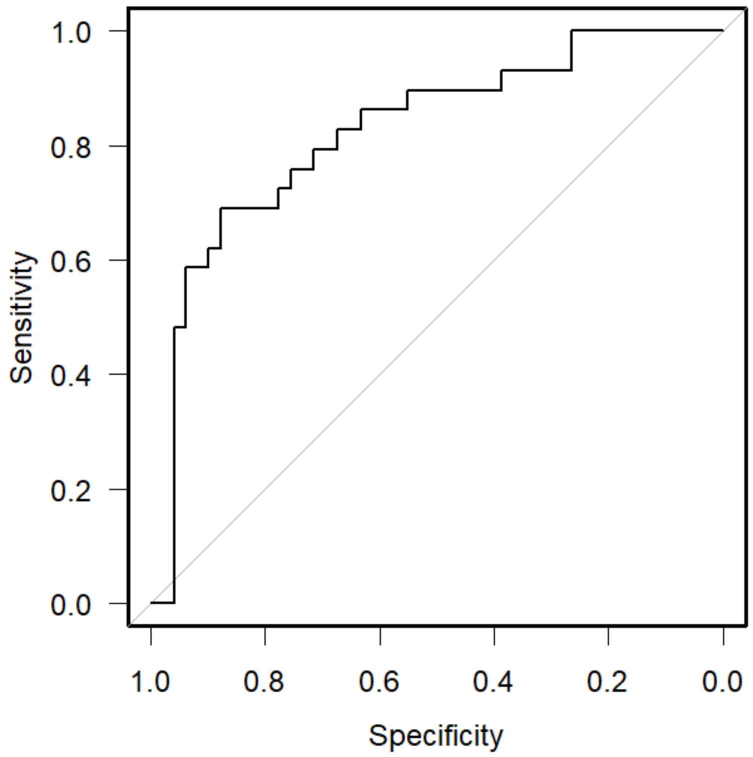

Supplementary Figure S1. Receiver-operating characteristic curve analysis showed an area under the curve of 0.825. A propensity score was calculated using logistic regression analysis based on age, sex, Eastern Cooperative Oncology Group performance status, Geriatric 8 level, and instrumental activities of daily living scores. After propensity score matching, the area under the receiver-operating characteristic curve was 0.825 (95% confidence interval 0.726–0.923).
